# IL-1 Inhibition May Have an Important Role in Treating Refractory Kawasaki Disease

**DOI:** 10.3389/fphar.2017.00163

**Published:** 2017-03-28

**Authors:** Perrine Dusser, Isabelle Koné-Paut

**Affiliations:** Department of Pediatric Rheumatology, Reference Centre for Autoinflammatory Diseases, Le Kremlin-Bicêtre Hospital, Assistance Publique-Hôpitaux de Paris, Paris-Sud University HospitalParis, France

**Keywords:** Kawasaki disease, vasculitis, pediatric, interleukin-1, coronary artery aneurysms, pediatrics, autoinflammatory disease

## Abstract

Kawasaki disease (KD) is an acute inflammatory vasculitis occurring in young children before 5 years and representing at this age, the main cause of acquired heart disease. A single infusion of 2 g/kg of intravenous immunoglobulins along with aspirin has reduced the frequency of coronary artery aneurysms from 25 to 5%. However, 10–20% of patients do not respond to standard treatment and have an increased risk of cardiac complications and death. The development of more potent therapeutic approaches of KD is an urgent need. Phenotypical and immunological similarities between KD and systemic juvenile idiopathic arthritis led to the hypothesis that KD could be considered as an autoinflammatory disease. New insights regarding KD’s pathogenesis have merged from the combination of genetic and transcriptomic data revealing the key role of interleukin-1 (IL-1) signaling in the pathogenesis of the vasculitis. Once activated, IL-1α and IL-1β trigger a local proinflammatory environment-inducing vasodilatation and attracting monocytes and neutrophils to sites causing tissue damage and stress. Both IL-1α and IL-1β have been shown to induce myocarditis and aneurysm formation in *Lactobacillus casei* cell-wall extract mouse model of KD; both being successfully improved with IL-1 blockade treatment such as anakinra. Treatment failure in patients with the high-risk inositol-triphosphate 3-kinase C genotype was associated with highest basal and stimulated intracellular calcium levels, increased cellular production of IL-1β, and IL-18, and higher circulating levels of both cytokines. Three clinical trials of IL-1 blockade enrolling KD patients are currently being conducted in Western Europe and in USA, they could change KD outcome.

## Introduction

Kawasaki disease (KD) is an acute inflammatory vasculitis of the medium- and small-sized arteries generally occurring in children under 5 years old. It was first described by Kawasaki, 1967 associated with the development of coronary artery aneurysms (CAA) or ecstasies in 15–25% of untreated children. Coronary lesions may lead to ischemic heart disease and sudden death. The etiopathology of KD remains unknown though it is widely accepted that it results in an important inflammation cascade triggered by unknown infectious or other stress trigger in a genetically predisposed individual. A single infusion of 2 g/kg of intravenous immunoglobulins (IVIGs) along with aspirin is the standard treatment for KD but not all children may respond, especially the youngest ones and those predisposed to develop CAA. Interleukin 1 (IL-1) cytokine has been shown to play a key role in the development of CAA leading to a potential use of IL-1 blockade in patients with KD.

## Kawasaki Disease

Classically, KD is diagnosed in the presence of high fever lasting for at least 5 days associated to at least four principal features (**Table 1**). No blood tests are available for diagnosis of KD, therefore, a clinical algorithm has been established and validated by the American Academy of pediatrics (**Table 1**) (Newburger et al., 2004). In some cases, KD diagnosis can be made at day 4 of illness in the presence of ≥4 principal criteria. Some patients have incomplete KD, especially infants ≤6 months. In this situation, KD diagnosis is challenging and should be looked for in infants with ≥7 days of fever without explanations, even though no KD clinical criteria are found. In this case, children should, therefore, undergo laboratory testing and, if any systemic inflammation is found, an echocardiogram should be performed (Newburger et al., 2004). Echocardiographic evaluation should be performed at the time of diagnosis, at 2 weeks and at 6–8 weeks after onset of the disease. More frequent echocardiographic evaluation is needed in children at higher risk (Newburger et al., 2004). Kawasaki disease vasculitis may occur outside the heart in other medium-sized vessel such as axillary, renal and femoral arteries somewhat difficult to distinguish from infantile periarteritis nodosa (Burns and Glodé, 2004).

**Table 1:** Kawasaki disease (KD) clinical algorithm (Newburger et al., 2004).

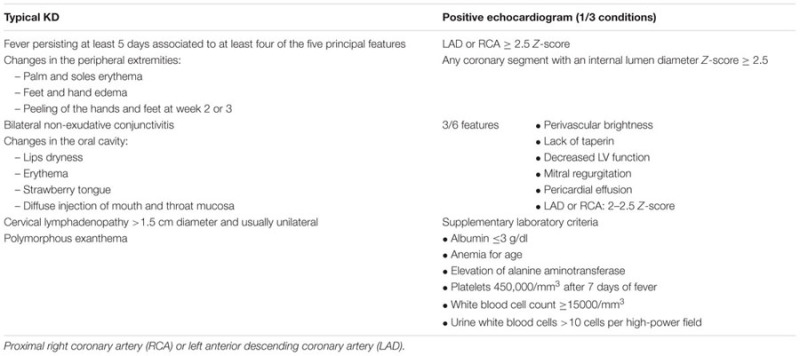

### Treatment in KD

A single infusion of 2 g/kg of IVIG along with aspirin has reduced CAA frequency from 25 to 5%. However, 10–20% of patients do not respond to standard treatment and have an increased risk of cardiac complications and death.

Corticosteroids (CS) as well as anti-tumor necrosis factor (TNF) agents are the two main treatments used in IVIG-resistant patients (Eleftheriou et al., 2014). Although there are no formal recommendations regarding optimal CS doses and duration (Chen et al., 2013), CS has not shown significant differences compared to an additional IVIG treatment in terms of preventing the development of CAA (Miura et al., 2008; Ogata et al., 2009). The elevated level of TNF-α in the sera of KD patients correlated with CAA development has led to the use of anti-TNF agent (Eleftheriou et al., 2014). The most frequently used is infliximab (IFX), a chimeric murine/human IgG1 monoclonal antibody that binds to TNF-α. This treatment has been administered in IVIG-resistant patients with success regarding fever and inflammatory parameters, however, with no differences regarding cardiac disease (Burns et al., 2005, 2008; Son et al., 2011). Other immunosuppressive agents have occasionally been used such as: cyclosporine, cyclophosphamide, methotrexate, and plasma exchange in resistant KD patients to IVIG, steroids, and anti-TNFα (Galeotti et al., 2016).

Although the use of different treatments has changed KD outcome, this disease is still lethal in certain cases. The individual prognostic factors are still poorly defined, and resistance to standard therapy represents a major risk of cardiac complications. Developing more efficient treatments and with a better action on cardiac involvement seems a priority. The phenotypical similarities between KD and systemic autoinflammatory disease (SAID) led researches to look at the role of inflammatory cytokines namely IL-1 in KD.

### Why to Use IL-1 Blockade in KD?

#### KD and Systemic Juvenile Idiopathic Arthritis (SJIA): Is There a Missing Link?

Kawasaki disease and SJIA represent a major cause of fever of unknown origin in young children and share intriguing similarities. Clinically, both diseases present with high fever, macular rashes, myalgia, arthralgia, and adenopathy although arthritis seems to be specific to SJIA (Lefèvre-Utile et al., 2014); for this reason, it is difficult to differentiate KD from early SJIA especially when KD is incomplete. Early age of presentation seems to favor KD. Moreover, cardiac abnormalities have been described, especially serositis as in many SAID. Unlike KD, SJIA coronary lesions are mild (essentially hyper echogenic coronaries) with favorable evolution; no CAA are described. Because of CAA risk in incomplete KD and need for early treatment, many patients with SJIA may be initially treated as KD, with IVIG and aspirin, but without efficacy (Lefèvre-Utile et al., 2014). Looking at laboratory findings, no differences can be seen. Both present elevated C-reactive protein, leukocytosis, thrombocytosis, hypoalbuminemia, anemia, and even macrophage activation syndrome (MAS) (Lefèvre-Utile et al., 2014). Assumptions have been made that these two systemic inflammatory disorders could share common triggering agents, susceptibility factors, or immunopathogenic pathways.

When looking at sera of KD and SJIA patients, inflammatory cytokines such as IL-1, IL-6, TNF-α, and interleukin-18 (IL-18) are increased. IL-18 being specifically higher in SJIA compared to KD patient (Mizuta et al., 2016). These phenotypical similarities between KD and SJIA along with the immunological features led to the hypothesis that KD could be considered as an SAID as SJIA and cryopyrin-associated periodic syndrome (CAPS) (Alphonse et al., 2016).

#### IL-1 Signature in SJIA and KD:

Inflammatory cytokines, especially IL-1β, has first been described as markedly increased in SAID such as CAPS and SJIA (Goldbach-Mansky, 2012). CAPS has allowed to understand the key role of IL-1 in the disease pathogenesis and showed striking response to IL-1-blocking therapies (Ter Haar et al., 2013). NLRP3 is a nod-like receptor (NLR) that is part of an inflammasome, which activates the caspase-1 (CASP1) and consequently the secretion of active IL-1β and IL-18 (Baroja-Mazo et al., 2014). *NLRP3* gene mutations result in constitutive activation of the NLRP3 protein and in an amplification loop of inflammation in which normal regulatory systems, i.e., ATP and second signal requirement are debrided, and where the pro IL-1β may act itself as a danger signal (Koné-Paut and Galeotti, 2015).

More recently, IL-1 has been shown to play a critical role in the pathogenesis of SJIA. Pascual et al. (2005) showed three major results. First, serum from SJIA patients induces the transcription of innate immunity genes including IL-1 in peripheral blood mononuclear cells (PBMCs) from healthy volunteers. Second, when activating PBMCs of SJIA patients, a large amount of IL-1β is released. Finally, they showed that, the use of recombinant IL-1 receptor antagonist (IL1-RA) (anakinra) allowed complete clinical remission in seven of the nine refractory-treated patients thus, emphasizing the central role of the innate immune system (IIS), and specifically, inflammasome-derived cytokines, in the pathogenesis of SJIA (Pascual et al., 2005).

As in systemic diseases, IL-1 seems to play a key role in the physiopathology of KD and more importantly in cardiac involvement for various reasons. Alphonse et al. (2016) showed a significant increased level of IL-1β, IL-18 and of their antagonists (IL-1RA and IL-18BP) in acute KD patients compared with age-matched control patients with viral or bacterial infections. Moreover, IL-1-induced inflammation has been shown to play a role in acute myocardial infarction and contributes to acute ischemic diseases. Indeed, IL-1 is known to enhance the expansion, differentiation and migration of antigen-specific CD8+ T cells as well as the induction of matrix enzymes source of major tissue damage. In the heart and brain, this inflammation can be fatal (Martinon and Tschopp, 2004). In KD, antigen-driven CD8+ T cells are known to infiltrate the coronary artery wall and contribute to the pathogenesis of CAA (Brown et al., 2001). The assumption appears all the more justified when looking at IVIG mechanism on inflammatory cytokines. In responsive KD patients treated with IVIG therapy, the level of pro-inflammatory cytokines (IL-1β, IL-6, and TNF-α) are decreased emphasizing immunoglobulin’s (IG) effect on the modulation of inflammatory cytokines namely on IL-1. Although the way IVIG acts is not perfectly understood, it is known to reduce CAA prevalence (Galeotti et al., 2010).

Interleukin-1 polymorphisms could be associated either to response or resistance to IVIG treatment (Weng et al., 2010). Interestingly elevated transcripts have been shown in IVIG-resistant KD patients, which carry the highest risk for coronary aneurysms (Fury et al., 2010). Increased transcript abundance of the neutrophil-associated calcium-binding proteins, S100A8 and A9, confirms the role of activated neutrophils in acute KD, as these proteins regulate adhesion of neutrophils and monocytes to the endothelial cell, a critical process in KD vasculitis. S100A8/9 proteins are elevated in patients who develop coronary aneurysms. The S100A8/9 heterodimer is known to activate the IL-1 receptor-associated kinase and the NF-_κ_B. S100A8/9 appears to be useful biomarkers for identifying IVIG-resistant patients. Other markers of endothelial cell activation CEACCAM1 (carcino embryonic antigen-related cell adhesion) and VEGF (vascular endothelial growth factor) have been detected in acute KD and may correlate with IGIV resistance and coronary vasculitis (Weng et al., 2010).

The role of IIS in the histopathology of KD has also been shown *in vivo* in mice.

### Mouse Model of CAA- and IL-1-signaling Pathways

A mouse model of coronary arteritis has been developed using intraperitoneal injection of *Lactobacillus casei* cell-wall extract (LCCWE). This mouse develops a focal, localized coronary arteritis that histopathologically mimics the coronary artery lesions found in human KD (Lehman et al., 1985). As in human CAA, the coronary lesions of LCCWE contains macrophages, activated dendritic cells, and T cells (Schulte et al., 2009). Moreover, the CAA in LCCWE mice responds to IVIG therapy as in KD children (Lehman, 1993). Although both innate and adaptive immunity have been shown as essential for the development of CAA in the LCCWE mouse model, IIS seems to play a key role. Two cytokines have been described as important in the development of CAA: NF-κB and IL-1. Rosenkranz et al. (2005) have pointed out the role of toll-like receptors (TLRs), a major sensor of IIS, in KD inflammation and therefore in CAA. In LCCWE, NF- NF-κB, an inflammatory cytokine, is activated and synthesized after activation of TLR-2 using a MyD88-dependent pathway (Rosenkranz et al., 2005). NF-κB activation coordinately controls both the innate and adaptive immune responses. To induce vasculitis in LCCWE mice, TLR2 are required as IL-1R signaling highlighting, amongst others, the importance of IL-1-signaling pathway in vasculitis (Rosenkranz et al., 2005). Both IL-1α and IL-1β have been shown to induce aneurysm formation in LCCWE mouse model of KD; aneurysm that are successfully improved with IL-1 blocker treatment such as anakinra (Schett et al., 2016). Similar successful results were reported in recalcitrant KD children using IL-1 blockade (Alphonse et al., 2016).

Lee et al. (2012) presented a mouse model of a knock-out LCCWE mouse (CASP1-/- and IL-1R-/-) in whom KD finally developed after injection of recombinant IL1-β protein. This mouse developed coronary arteritis, which could be prevented by injection of the IL-1 receptor antagonist (IL-1RA): anakinra, during 3–5 days. Using the LCWE mouse model, a logical progression of experiments demonstrated that (i) bone marrow-derived macrophages secrete high levels of IL-1β and TNFα; (ii) IL-1β is processed from pro-IL-1β by CASP1 through the NLRP3 inflammasome; (iii) exogenous treatment with IL-1β recreates the inflammatory phenotype in CASP1 deficient mice; and (iv) IL-1R-deficient mice or mice treated with the recombinant IL-1RA, anakinra fail to develop the arteritis lesions. Of particular note, only blockade of IL-1β, but not blockade of TNF-α, reduced the myocarditis in the LCWE-injected mice (Burns, 2012; Lee et al., 2012). A recent case report showed a dramatic effect on rescuing a life-threatening case of relapsing KD (Cohen et al., 2012).

### Genetics: IL-1 Pathway and Calcium Signaling

Finally, analysis of the whole-genome expression profile of acute KD patients has pointed out the importance of IL-1β activation in KD inflammatory profile by showing the link between calcium concentration and inflammasome.

Inositol-triphosphate 3-kinase C (ITPKC) is a candidate gene located on chromosome 19q13.2 whose CC genotype is implicated as a determinant of both disease susceptibility and outcome in KD. ITPKC phosphorylates inositol 1, 4, 5-triphosphate (IP3) to inositol 1, 3, 4, 5-tetraphosphate (IP4), therefore, regulating the calcium response to extracellular signals. At the same time, NLRP3 inflammasome has been shown to be dependent of both extracellular and intracellular calcium concentration ([Ca^2+^]i). Amazingly, ITPKC CC genotype is associated with both highest basal and stimulated [Ca^2+^]i levels and increased amounts of NLRP3 protein compared with other genotypes at baseline. These findings, allowed making the hypothesis of a link between the calcium level and the activation of NLRP3 in ITPKC CC genotype leading to an excess of IL-1 secretion as in SAID. Moreover, ITPKC CC genotype is associated with failure of IVIG therapy (Alphonse et al., 2016). This emphasizes the fact that phenotypic similarities between KD and AID are anchored by the common immunobiological processes associated with inflammasome activation.

### Experience of IL-1 Blockade in KD Patients

Nowadays, three IL-1 blockades have been approved: anakinra, rilonacept, and canakinumab. Anakinra (Kineret^®^) was the first IL-1 blockade agent administered initially in rheumatoid arthritis (1993) and is now used in numerous diseases such as hereditary SAIDs (Schett et al., 2016). It is an IL1-RA blocking both IL-1α and IL-1β (Carter et al., 1990). In 2008, rilonacept (Hoffman et al., 2008), a soluble IL-1 decoy receptor, that neutralizes either IL-1α or IL-1β, received US Food and Drug Administration (FDA) approval in CAPS patients, and Canakinumab (Ilaris^®^) in 2009 (Chakraborty et al., 2012). The latter is a humanized monoclonal antibody that specifically blocks IL-1β (Dinarello et al., 2012). In pediatrics, only anakinra (≥8 months and 10 kg) and canakinumab (≥24 months and ≥7.5 kg) have FDA and European Medicines Agency (EMA) approval for CAPS disease. These IL-1 blockades are safe and well tolerated with a low-adverse event rate (Rossi-Semerano et al., 2015). Anakinra is preferred for it has a remarkable record of safety with over 150,000 patients treated daily for over 10 years (Bresnihan et al., 2004; Fleischmann et al., 2006). In addition, drug level significantly drops 1 h after discontinuation of treatment (Dinarello et al., 2012).

In SJIA, the three IL-1 blockers have been tested so far and were proven as effective and safe, although only canakinumab is currently approved for use (Giancane et al., 2016). In addition, anakinra has been demonstrated as efficient in severe SJIA with MAS (Miettunen et al., 2011) a severe complication that can occur in up to half of SJIA patients. In KD, MAS is probably a frequently under-recognized complication situation which could benefit from IL-1 blockers (Wang et al., 2015).

For now, two case reports showing promising results with anakinra in severe KD patient have been published. The first one is an 11-week-old Caucasian female that presented with severe KD complicated by MAS. Diffuse enlargement of the entire coronary artery system was revealed by echocardiogram. IVIG, aspirin and CS were inefficient. High doses of anakinra (3 mg/kg/dose, twice daily for 3 days) were introduced at day 6 because of bad clinical outcome and biological signs of MAS. IFX and methylprednisolone were added at day 9 because of cardiac failure despite favorable clinical and biological course. The evolution was favorable allowing CS to be tapered off over 10 days following discharge and under anakinra over the next 5 months. At 8 months, the echocardiogram was normal (Shafferman et al., 2014).

The second one is a 2-year-old boy diagnosed with KD who developed secondarily cardiac failure (shortening fraction of 20%) without CAA under IVIG (2 g/kg) treatment. A second IVIG perfusion was administered as well as multiple methylprednisolone pulses with little effect and worsening of cardiac involvement. Extracorporeal membrane oxygenation was performed from day 14 until day 17 and subcutaneous anakinra (1 mg/kg/day) was introduced at day 18 until day 24 with success. Relapse was seen three days after anakinra’s last injection with progression to giant CAA. Anakinra was therefore reinitiated for 6 weeks with normalization of the coronary lesions at 6 months (Cohen et al., 2012).

### What Could Be the Place of Anti-IL1 in the Current Standard of KD Treatments?

Considering current knowledge, it seems reasonable to use IL-1 blockade in resistant KD with CAA before IFX which has not proven its efficiency in coronary disease. A new approach could be the early use of IL-1 blockade associated with CS in patients at high risks of severe KD depending on validated risk scores, in Japanese patients. Apart from IVIG’s, anti-IL1 are the only therapies that have proven their effect on CAA. It should be considered whether their use should not be generalized to all patients. Indeed, IL-1 blockers seem to better prevent CAA development than IVIG, especially if used at diagnosis. In this idea three clinical trials of IL-1 blockade enrolling KD patients are currently being conducted in Western Europe and in the US (**Table 2**) (Burns et al., 2016). Their conclusion may help to better define, in the future, the place of IL-1 blockade in KD treatment in association or in replacement of IGIV and CS.

**Table 2:** Clinical trials of IL-1 blockade enrolling KD patients conducted in Western Europe and in USA (Burns et al., 2016).

Name	Type of trial	IL-1 blockade/Doses	Population KD patients	Objective	Time
Kawakinra trial (Europe) (Eudract Number: 2014-002715-41)	Phase IIa, multi-centered trial	Anakinra: 2 mg/kg/day Dose can be increased by 2 mg/kg/24 h if persistent or recrudescent fever (max 6mg/kg/d)	Children: 8 months (≥10 kg)–18 years Screened: 4th–13th day of fever	Primary end points Efficacy and safety of anakinra Secondary objectives Effects of anakinra on coronary artery Disease activity and inflammation biomarkers	14 days of treatment
Anakid trial (USA) (clinicaltrials.gov#NCT02179853)	Phase I/IIa study: Two-centered and dose escalation trial	Anakinra: 2, 4 or 8 mg/kg Persistent or recrudescent fever after ≥36 h and <7 days following the end of intravenous immunoglobulin (IVIG) infusion	Children (≥8-M -old) with acute KD and with coronary artery (*Z*-score ≥3.0 in the RCA or LAD) abnormalities	Safety, tolerability, and pharmacokinetics of anakinra	2–6 weeks
Canakinumab trial (Europe)	Phase II trial: Two-arm, multi-centered and carried out in seven European countries	Canakinumab: 6 mg/kg IV Group 1: Complete fever resolution Canakinumab (1 or 2 SC injections) at 4 and 8 weeks Depending on the clinical and CRP course Group 2 Fever remains after 48–72 h of canakinumab IVIG	Naïve KD patients or IVIG-resistant KD patient	The presence or absence of fever will be looked-for.	

*IVIG, intravenous immunoglobulin; RCA: right coronary artery; LAD, left anterior descending coronary artery; SC, subcutaneous.*

## Conclusion

Kawasaki disease clinical and immunological features mimic SAID. These similarities have allowed looking at new inflammatory cytokines such as IL-1. Better understanding of IL-1 involvement in KD and specifically in CAA with the use of IL-1 blockers, has brought hope for resistant and severe patients. Doses and time to introduce IL-1 therapy has still to be defined. Another challenge is the need to better define patients with a higher risk of CAA, allowing better medical care and the use of new treatment strategies. We hope that results of clinical trials using IL-1 blockade will allow to better understand the respective roles of IL-1α and β, and to pursue with phase III trials.

## Author Contributions

Both the authors had a substantial contribution to the work. PD wrote the first draft. PD and IK-P were involved in drafting the article or critically revising it for important intellectual content and approved the final version to be published.

## Conflict of Interest Statement

The authors declare that the research was conducted in the absence of any commercial or financial relationships that could be construed as a potential conflict of interest.
